# Environmental justice implications of arsenic contamination in California’s San Joaquin Valley: a cross-sectional, cluster-design examining exposure and compliance in community drinking water systems

**DOI:** 10.1186/1476-069X-11-84

**Published:** 2012-11-14

**Authors:** Carolina L Balazs, Rachel Morello-Frosch, Alan E Hubbard, Isha Ray

**Affiliations:** 1 Energy and Resources Group, University of California, Berkeley, CA, 94720, USA; 2 School of Public Health, University of California, Berkeley, CA, 94720, USA; 3 Department of Environmental Science Policy and Management, University of California, Berkeley, CA, 94720, USA

**Keywords:** Revised arsenic rule, Arsenic, Drinking water, Social disparities, Environmental justice, Water systems, Safe drinking water act, Exposure

## Abstract

**Background:**

Few studies of environmental justice examine inequities in drinking water contamination. Those studies that have done so usually analyze either disparities in exposure/harm *or* inequitable implementation of environmental policies. The US EPA’s 2001 Revised Arsenic Rule, which tightened the drinking water standard for arsenic from 50 μg/L to 10 μg/L, offers an opportunity to analyze both aspects of environmental justice.

**Methods:**

We hypothesized that Community Water Systems (CWSs) serving a higher proportion of minority residents or residents of lower socioeconomic status (SES) have higher drinking water arsenic levels and higher odds of non-compliance with the revised standard. Using water quality sampling data for arsenic and maximum contaminant level (MCL) violation data for 464 CWSs actively operating from 2005–2007 in California’s San Joaquin Valley we ran bivariate tests and linear regression models.

**Results:**

Higher home ownership rate was associated with lower arsenic levels (ß-coefficient= −0.27 μg As/L, 95% (CI), -0.5, -0.05). This relationship was stronger in smaller systems (ß-coefficient= −0.43, CI, -0.84, -0.03). CWSs with higher rates of homeownership had lower odds of receiving an MCL violation (OR, 0.33; 95% CI, 0.16, 0.67); those serving higher percentages of minorities had higher odds (OR, 2.6; 95% CI, 1.2, 5.4) of an MCL violation.

**Conclusions:**

We found that higher arsenic levels and higher odds of receiving an MCL violation were most common in CWSs serving predominantly socio-economically disadvantaged communities. Our findings suggest that communities with greater proportions of low SES residents not only face disproportionate arsenic exposures, but unequal MCL compliance challenges.

## Background

Arsenic in drinking water is linked to skin, lung, bladder and kidney cancers [1-3]. The most common exposure pathway is consumption of groundwater containing arsenic [4]. Many epidemiological studies examining health effects of arsenic in drinking water have been conducted in areas with extremely high levels (i.e., > 100 μg As/L)—such as Argentina, Bangladesh and Taiwan. But high concentrations (i.e., 50–100 μg As/L) also occur in the U.S, especially in western regions such as Utah, Nevada, Arizona and California [5-8]. Here, arsenic in groundwater is generally naturally occurring, but can also derive from agricultural activities including pesticide application and industrial uses (e.g. wood treatment) [4,9]. In California’s San Joaquin Valley, arsenic can reach elevated concentrations due to mobilization caused by agricultural activities. In particular, irrigation and drainage enhance arsenic releases, while high evapotranspiration rates can concentrate arsenic in surface water and shallow groundwater [4,10,11].

In 2001, on the basis of epidemiologic evidence and cost-benefit considerations [12] the U.S. Environmental Protection Agency (EPA) issued the Revised Arsenic Rule, reducing allowable arsenic concentrations in drinking water from 50 μg/L to 10 μg/L. The revision of this drinking water standard came with much debate. Critics of the standard argued that there was uncertainty in the risk assessment, and that the cost-benefit analyses overestimated benefits in relation to costs of compliance. Ultimately, however, the EPA’s Science Advisory Board and the National Research Council (NRC) concluded that the science was sufficient to warrant a more health-protective standard [12-14].

The revised rule elicited considerable discussion regarding equity considerations for small water systems [15,16]. Of the estimated 5.5% of community water systems that were expected to be affected by the Revised Arsenic Rule, nearly 97% were small systems serving fewer than 10,000 customers [17]. Benefit-cost analyses concluded that although there would be a net benefit for households, the average annual compliance costs for residents served by smaller systems would be much greater. Recognizing this discrepancy, the US EPA extended the compliance date by two years for systems serving fewer than 10,000 customers, assessed alternative affordable technologies for small systems and focused on analyzing additional impacts that would be felt by these systems [14]. Effective in 2002, the Revised Arsenic Rule required all public water systems to comply with the new standard by January 23, 2006 [14].

Besides these scale-related considerations, however, little attention was given to other potential social disparities that could arise in, for example, exposure to arsenic, or the types of small systems that would be able to comply with the revised standard. In response, several environmental justice-oriented studies explored potential inequities in exposure to arsenic [18,19] and in enforcement of the arsenic standard [5]. Generally, these studies focused on two types of distributional issues: (1) disparities in environmental harms, such as exposure to contaminants, or disparities in health outcomes, and (2) disparities in the inequitable implementation of policies and programs, including access to federal funds or capacity to comply.

Attention to both components of environmental justice is certainly warranted. We argue, however, that a joint focus -- on compliance challenges as well as exposure to contaminants -- is most helpful for understanding the health and social implications of drinking water policies, including the Revised Arsenic Rule. Quantifying a water system’s compliance with the arsenic MCL is important to know which systems are in violation, and to consider whether they are equipped to comply. This “compliance burden” allows for an exploration of whether certain groups or communities have unequal abilities in the capacity to meet the standard. Quantifying exposure levels and their distribution is important, given known health risks at levels even below the new standard. Thus this study employs what we term a “joint burden analysis,” to analyze the environmental justice implications of compliance capacity and exposure related to arsenic contamination. Together, these analyses provide a picture of the joint burdens that water systems and residents may face.

We applied a cross-sectional analysis of social disparities related to the Revised Arsenic Rule. We conducted our study in California’s San Joaquin Valley, one of the poorest regions in the country with some of the most contaminated drinking water sources in California [20], including high nitrate and high arsenic levels [21]. We focused on community water systems (CWSs), which are public water systems that serve at least twenty five customers or fifteen service connections year-round [22]. We hypothesized that CWSs serving a higher proportion of minority or lower socioeconomic status (SES) residents have a higher odds of non-compliance with the revised arsenic standard and that these CWSs serve drinking water with higher levels of arsenic.

Our analysis provides two contributions to the arsenic and drinking water literature. By assessing exposure disparities and compliance burdens at the time of the enactment of the Revised Arsenic Rule, we assess the potential exposure and compliance disparities that existed but were not fully incorporated into policy assessments. Secondly, we consider the compliance challenges that CWSs could face moving forward, broadening the discussion of policy implementation issues that must be considered by drinking water regulators and the US EPA.

Given the U.S. EPA’s renewed discussion of the impact of the Revised Rule on small systems, and on how to help small systems achieve compliance, the results of this study are timely for policy circles as well. For example, the U.S. EPA recently convened a working group on arsenic in small water systems to provide input on barriers to the use of point-of-use and point-of-entry treatment units, as well as alternative affordability criteria that pay particular attention to small, rural, and lower income communities [23]. Our study’s quantitative analysis of the distribution of exposure and compliance burdens therefore adds to the environmental justice literature and informs these policy discussions.

## Methods

### Sample selection and selection of point-of-entry sources

We selected CWSs located in California’s San Joaquin Valley that were actively operating between 2005 and 2007, had at least one source with a geographic coordinate that could be used to estimate customer demographics, and had at least one active point-of-entry source with an arsenic sample reported during this period. These selection criteria resulted in a slight under-representation of smaller systems (i.e., < 200 connections) in our final sample (see Additional file 1: Table A1). Our time period represents one full compliance period under the SDWA, in which each CWS should have taken at least one arsenic sample [24].

Point-of-entry sources are those that directly enter the distribution system. We selected two types of point-of-entry sources: (1) sources in active use that had no arsenic treatment, or that treated for contaminants other than arsenic, and (2) treatment plants in active use that potentially treated for arsenic (Additional fle 2: Figure A1). We used the California Department of Public Health’s (CDPH) Permits, Inspections, Compliance, Monitoring and Enforcement (PICME) database [25] to identify source types, their location in relation to the distribution system, and their possible treatment techniques. We confirmed the existence of arsenic treatment technologies with state and county regulators.

For the six CWSs with confirmed arsenic treatment plants that were in use during the study period, we used all point-of-entry sampling points prior to installation of treatment, and only sampling points from treatment plants after the installation date. For CWSs with no confirmed arsenic treatment, we selected systems where either all point-of-entry sources were labeled as untreated, or all point-of-entry sources were labeled as having treatment. In practice a CWS may have both treated and untreated sources. But because the CDPH databases did not allow us to accurately ascertain whether untreated sources entered the distribution system if treated sources were also available, we conservatively selected CWSs in this manner. We tested the sensitivity of this decision by comparing regression results using our final sample to results using all CWSs. Our final sample included 464 of the 671 CWS active in the Valley from 2005 to 2007.

### Outcome measures and independent variables

In order to assess compliance with the Revised Arsenic Rule (i.e., MCL violations) and exposure burdens, we conducted two main sets of analyses: one focused on MCL violations, the other on exposure. Specifically, for each CWS, we derived four main outcome measures: (1) officially recorded arsenic MCL violations, (2) average system and source-level arsenic concentrations, (3) population potentially exposed to arsenic, and (4) water quality samples of arsenic concentrations at point-of-entry to the distribution system. We used the first measure to analyze compliance. We used the second two measures to derive descriptive exposure statistics and run sensitivity analyses. We used the fourth measure as the outcome variable in a linear regression model. We calculated average arsenic measures because (1) the MCL for arsenic is assessed using running annual average of arsenic concentrations for water systems; and (2) this MCL is based on a consideration of long-term chronic exposure making the average concentration of arsenic a suitable metric.

#### Arsenic MCL violations

The key outcome for our compliance analysis was officially recorded arsenic MCL violations derived from the PICME database. We created a binary variable indicating whether a system had received at least one MCL violation during the study period. This measure helped control for bias that could occur because CWSs with higher arsenic levels are required to sample more frequently [26], thereby increasing the probability that they would receive more MCL violations.

#### Average system and source-level arsenic concentrations

To estimate arsenic concentrations in the distribution system we used arsenic water quality sampling data for the selected point-of-entry sources from CDPH’s Water Quality Monitoring database [27] (Additional file 2: Figure A1). Previous studies have noted the benefit of using such publicly available water quality monitoring records as an alternative to costly tap water samples [28]. Using these data points, we derived the average arsenic concentration served by each CWS for the entire compliance period. We calculated this by averaging the average source concentrations for each system during our time period. As in previous studies [5,19], we assumed average system-level concentrations represent the average arsenic concentration in water served to residents. We also calculated each CWS’s yearly average arsenic concentration to conduct sensitivity analyses. Because we did not have flow measurements for individual sources, we assumed that each point-of-entry source contributed independently, constantly and equally, to a CWS’s distribution system, regardless of season. For sampling points below the detection limit, we took the square root of the detection limit as a proxy for the arsenic concentration [29].

We categorized source-level and system-level averages into three concentration categories defined in relation to the revised arsenic rule (> 10 μg As/L) and the old rule (> 50 μg As/L): (1) < 10 μg As/L (“low”), (2) 10–49.9 μg As/L (“medium”), and (3) ≥ 50 μg As/L (“high”). In addition, we used average source and system-level concentrations to create binary variables that we used in bivariate analyses. Here, average levels were coded as 1 (≥ 10 μg As/L), or 0 (< 10 μg As/L).

#### Potentially exposed population

Using a previously developed method [30] described in Balazs et al. [31], we computed the population potentially exposed to the three aforementioned exposure categories. The approach to calculate the potentially exposed population (PEP) for the high-arsenic category is summarized by the following equation:

(1) $$PEP_{h}=\sum_{i=1}^{464}\left(X_{i}\times s_{\mathrm{ih}}/S_{\mathrm{it}}\right)$$

where *X*_*i*_ is the total population served in CWS *i*; s_*ih*_ is the number of sources for CWS *i* with average arsenic concentrations classified as high (*h)*; and *S*_*it*_ is the total number of point-of-entry sources for CWS *i.* To calculate the PEP for the low (*l)* or medium (*m)* categories, we replaced s_*ih*_ with s_*il*_ or s_*im*_, respectively. We used PICME data on the number of people served by each CWS to calculate the population size. If the number of customers served by a CWS was not available from the PICME database, we used information from the Water Quality Monitoring database. To estimate counts of potentially exposed individuals according to demographic characteristics (e.g. race/ethnicity) we multiplied the PEP in each arsenic category for each CWS by the estimated proportion of customers in each demographic subgroup for the CWS (e.g. 50% people of color), and then summed these counts across all CWSs for each arsenic category.

#### Concentration of arsenic at point-of-entry

Arsenic sampling data for each point-of-entry source were used as the outcome variable in our regression model, as described under “Regression Model” below.

### Analyses

#### Compliance analyses

We used our binary arsenic MCL violation variable to analyze whether CWSs with higher fractions of people of color or lower SES faced greater compliance violations. Because only 34 CWSs had at least one MCL violation we did not have enough outcomes to use multivariate regression techniques. Instead we ran Fisher’s Exact tests for contingency tables, comparing the presence of at least one MCL violation to CWSs with high or low levels of our variables of interest (i.e. race/ethnicity or homeownership). To determine the threshold for high and low levels of race/ethnicity (i.e., percent people of color) or homeownership rate we used the median value of these variables.

To consider the impact of under- or mis-reported violations, we ran sensitivity analyses in which we replaced official MCL violations with the number of CWSs with any source whose average yearly arsenic concentrations exceeded the MCL during the study period, and the number of CWSs with any source whose compliance period average exceeded the MCL. This allowed for an approximation of whether a system may have exceeded the MCL (and so should have been issued an MCL violation) since arsenic MCL violations are based on a running annual average [26]. Thus these sensitivity analyses should capture differences due to MCL exceedances that went under-reported.

#### Exposure analyses

To assess the relationship between demographics of customers served by CWSs and potential exposure, we first examined the demographic characteristics of the population potentially exposed to three different arsenic levels, and additional characteristics of the systems at those levels. To further analyze the relationship, we used our binary variables for average system-level arsenic concentrations to conduct Fisher’s Exact tests.

Finally, we examined the relationship between system-level demographics and arsenic levels using our continuous measure of arsenic concentrations. We used a linear cross-sectional regression model with robust standard errors to account for clustering. To derive the inference, we clustered outcomes at the CWS-level (i.e. point-of-entry arsenic concentrations measured on a given day for a given source). Our final model reported sandwich-type robust standard errors [32] that allowed for arbitrary correlation, including correlation within CWS units. The a priori selected model controlled for known or hypothesized potential system-level confounders.

The model’s outcome variable, Y_*ijk*,_ was arsenic concentration for the *i*^th^ water system, the *j*^th^ source in system *i,* on day *k* (since January 1^st^, 2005). While arsenic samples from individual sources were our outcome measurements, the CWS was the primary unit of analysis, consistent with other calculations above. Our final model did not re-weight CWSs with more samples; thus systems with more measurements contributed more to the estimates. We addressed this issue by stratifying by system size to see if smaller CWSs (with fewer samples) had a different effect on water quality than larger CWSs.

Key independent variables were the percentage of people of color served by CWSs (referent category non-Latino whites) and percent home ownership in each CWS. Home ownership rate is a proxy metric for wealth and political representation [33]. We used this SES measure as an indicator of the economic resources available to a water system to mitigate contamination [34]. Race/ethnicity and home ownership data were derived from the 2000 U.S. Census, measured at the CWS-level, and assumed to be constant for all three years [35]. Since CWS service areas do not follow Census boundaries we used a spatial approach in Geographic Information Systems (GIS) to estimate demographic variables for each CWS. In brief, for each CWS, we estimated a population-weighted average of each variable across all block groups that contained sources for the CWS. This value was used to derive a percent estimate of demographic characteristics (e.g. 50% homeownership) served by that CWS [31].

We controlled for other potentially confounding water system characteristics including: source of water (ground water or groundwater and surface water versus surface water alone); system ownership (public, privately owned and not regulated by the Public Utility Commission (PUC), with private PUC-regulated as referent category); geographic location (Valley floor and foothills, with mountains as referent category); season (summer/fall or winter/spring); year of sampling (2006 and 2007, with 2005 as referent category); and number of service connections (< 200 or ≥ 200 connections). We determined ownership structure by combining data in PICME with data from the PUC’s list of regulated systems. We obtained all other characteristics from PICME. With the exception of year and season, all covariates were measured at the water system level.

We stratified by system size to assess if demographic effects on water quality might be stronger among smaller systems, and to test the hypothesis that scale alone explains water quality. We used number of connections as a threshold for small versus large CWSs, where those with fewer than 200 connections are considered “small” [26]. We used our final model to estimate the amount of arsenic contamination attributable to the proportion of the population that are homeowners by predicting expected values for each observation if percent homeownership equaled 100%, as described by Greenland and Drescher [36]. All statistical analyses were conducted using Stata v10 (College Station, Texas). We used Stata’s cluster command to derive robust standard errors.

## Results

### Descriptive statistics

The 464 CWSs in our study sample served 1.134 million people, representing 37% of the total population served by CWSs between 2005 and 2007, and 69% of all CWSs active through 2007 (Table 1). The mean percentage of people of color served by each CWSs was 39% [inter-quartile range (IQR), 16-57%]. The mean percent of homeownership was 70% (IQR, 60-81%). The yearly average arsenic concentration in 2005, 2006 and 2007 was 7.0 μg/L (median = 3 μg/L) 7.9 μg/L (median = 2.5 μg/L), respectively and 6.8 μg/L (median = 3 μg/L), respectively. Approximately 12% of samples were below the detection limit.

**Table 1:** Characteristics of community water systems (CWSs) in study sample compared to all CWSs in study region with geographic coordinates, 2005-2007, San Joaquin Valley, CA

**Variable of interest**	**Active CWS with geographic coordinates n = 644**	**CWS in study: active, w/ coordinates and arsenic samples n = 464**	**CWS in study: < 200 connections n = 324**	**CWS in study: ≥ 200 connections n = 140**
**Total population (count)**	3,037,785	1,134,017	49,340	1,084,677
**Population Characteristics (%)**				
People of Color (Latinos and Non-Latinos)	53	55	38	56
White population	47	45	62	44
Population above poverty^a^	57	54	60	54
**Water System Characteristics (%)**				
Mean People of Color	42	39	35	50
Mean Home Ownership	67	70	72	67
Population served (mean/median)	4,717/163	2,444/180	152/100	7,748/2537
Incorporated ^b^	10	9	1	29
< 200 Connections	72	70	100	0
Groundwater Alone (GW)^c^	88	92	95	87
GW and surface water^c^	7	4	2	9
Publicly owned^d^	32	32	13	75
Privately owned non-PUC reg.^d^	60	61	80	16
**Water Quality Characteristics**				
Min-Max; mean (μg As/L)	NA	0-158; 6.0	0-158; 6.2	0-42; 57
IQR (μg As/L)	NA	1.4, 6.3	1.4, 6.2	1.4, 7.3
CWS with ^3^1 As MCL Viol	44	34	15	19

*NA* not applicable because not all active sources had arsenic samples, *IQR* interquartile range.

^a^ Above 200% the poverty level; ^b^ A water system that serves a city that is a legally recognized municipal corporation with a charter from the state and governing officials that is incorporated, as opposed to a water system that serves an unincorporated area; ^c^ Reference group=surface water only; ^d^ Reference group=privately owned and Public Utility Commission (PUC) regulated or unknown.

**Figure 1:**
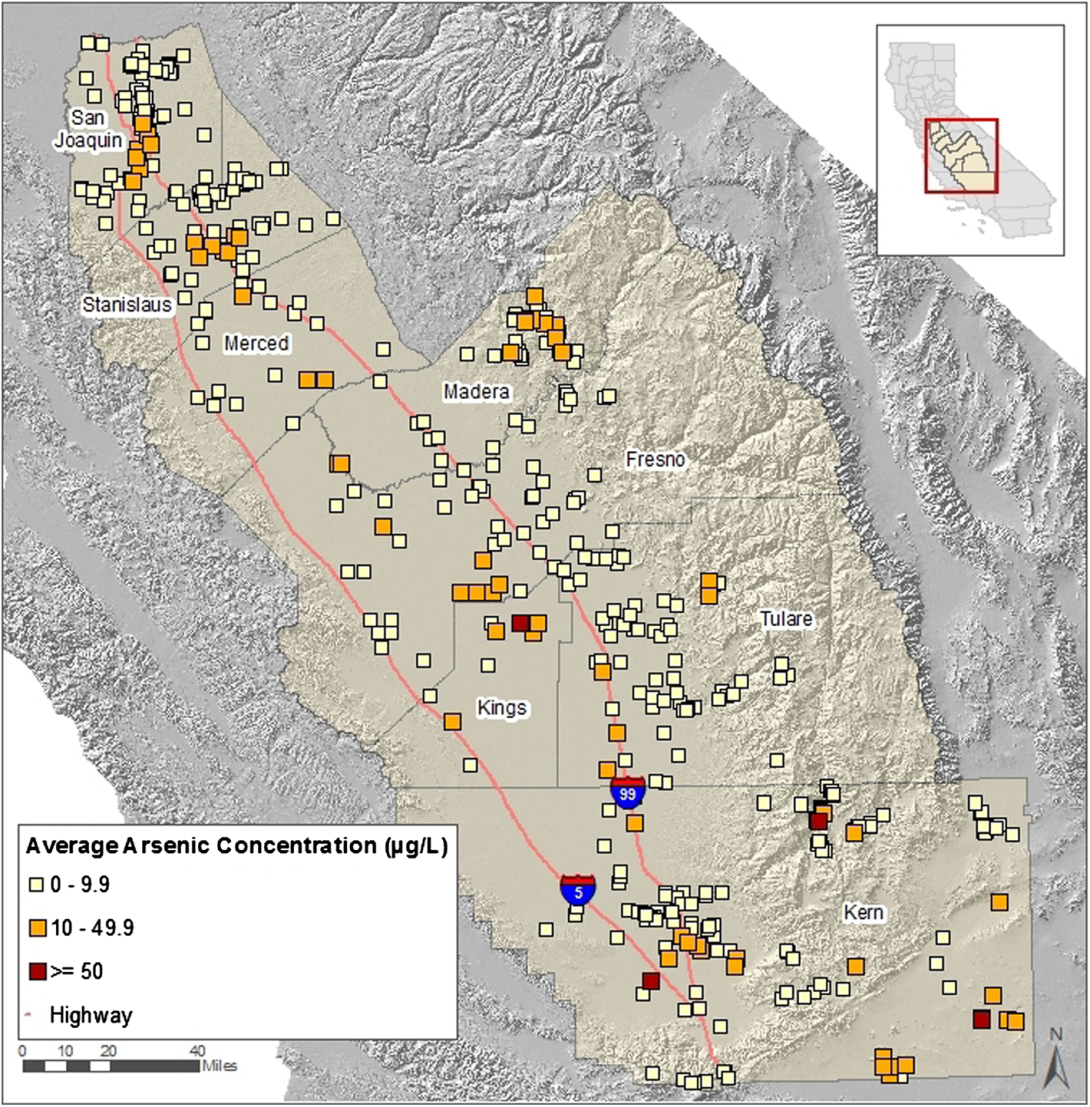
**Average arsenic concentration**^**a **^**of community water systems (CWS **^**b,c**^**) in study sample, (n = 464), 2005–2007.**^a^ Estimate based on average of each point-of-entry source’s average concentration; ^b^ Sources of data: CDPH Water Quality Monitoring and PICME databases (CDPH 2008a,b); ^c^ Approximate location of CWSs are depicted, but not true boundaries. Due to close proximity of some CWSs, map partially covers some CWSs.

Nearly 15% of all CWSs in the sample had average arsenic concentrations between 10 and 50 μg As/L, and were therefore affected by the revised standard (Table 2). Among these, 66% had fewer than 200 connections, and 86% had three active wells or less. For each CWS with average arsenic in this range, the average percentage of a CWS’s sources that exceeded the revised MCL was 87% (Table 2). Less than 1% of CWSs had average levels greater than 50 μg As/L. Among these, all had fewer than 200 connections. CWSs west of Highway 99 and in the central portion of the Valley had higher arsenic levels, as did some areas in the foothills and in southeastern Kern County (Figure 1).

**Table 2:** Characteristics of community water systems (CWSs) at three average arsenic levels, 2005–2007, San Joaquin Valley, CA

	**Average arsenic concentration**		
**CWS characteristics**	**< 10 μg/L**	**10-49.9 μg/L**	**≥ 50 μg/L**
% CWS	84.5	14.6	0.9
Mean Population Served (median)	2496 (180)	2277 (200)	127 (64)
% Privately owned, non-PUC Regulated	61	59	100
% < 200 Connections	70	66	100
Range of Mean Arsenic (μg As/L)	0-9.9	10.1-41.7	59.5-158
Mean μg As/L (Median)	3 (2)	19 (16)	97 (85)
Mean % of Sources > MCL (IQR)	.1 (0,0)	87 (75, 100)	100 (100, 100)
CWSs with arsenic treatment plant	2	4	0

*IQR* Interquartile range, *PUC* Public Utility Commission.

Of the population served in our sample, approximately 14% was potentially exposed to arsenic levels over 10 μg/L MCL (Table 3). Of the population potentially exposed to 10–50 μg As/L, 61% were people of color (i.e. Latinos and non-Latino people of color). This is higher than the corresponding percentage in the entire study sample (i.e., 55%, Table 1).

**Table 3:** **Demographic profile of potentially exposed population (PEP **^**a**^**) by average arsenic levels, 2005–2007, San Joaquin Valley, CA**

**Population characteristics**	**Average arsenic concentration**		
	**< 10 μg/L**	**10-49.9 μg/L**	**≥ 50 μg/L**
% Total Population (1,134,017)	86.1	13.7	0.2
% People of Color^b^	54	61	24
% Non-Latino White	46	39	76

^a^ Per water system, PEP = population count of demographic of interest x (# of sources in one of three arsenic level/total # of sources sampled). PEP displayed in table is equal to sum across all water systems. This value can also be interpreted as the estimated number of people served water at this level. ^b^ People of color refer to both Latino and non-Latino people of color.

### Statistical analyses

#### Compliance analyses: MCL violations

Thirty-four CWSs, serving 151,391 people, received at least one arsenic MCL violation during the study period. Of these, 31 had average system-level arsenic concentrations over 10 μg As/L and 3 had average concentrations of 8, 8.8 and 9.9 μg As/L. CWSs serving higher percentages of homeowners had a 67% lower chance of having at least one MCL violation (Table 4). CWSs serving higher percentages of people of color had a 260% higher chance of having at least one MCL violation. Sensitivity analyses in which we used average source-level concentrations were consistent, yielding results of similar strength and direction (see Additional file 3: Table A2).

**Table 4:** Fisher’s exact tests and related odds ratio (OR) for maximum contaminant level (MCL) violations, 2005–2007, San Joaquin Valley, CA

**Variable of interest**	**≥ 1 MCL violation**	**No MCL violation**	**OR (95% CI)**	**P-value**
High % Homeownership	12	269	.33 (.16, .67)	.003
Low % Homeownership	22	161		
High % People of Color	24	207	2.6 (1.2, 5.4)	.01
Low % People of Color	10	223		

Fisher’s Exact test compares high and low category for variable of interest, where threshold is determined by median value across all CWS, and includes related odds ratio. Test compares demographics in community water systems that received at least one MCL violation to those with zero violations.

#### Binary measure of exposure

CWSs serving higher percentages of homeowners had a 57% lower chance of having average arsenic levels above the revised MCL (Table 5). CWSs serving higher percentages of people of color had a 130% higher chance of having average arsenic levels above the revised MCL.

**Table 5:** Fisher’s exact tests and related odds ratio (OR) for average arsenic level, 2005-2007, San Joaquin Valley, CA

**Variable of interest**	**≥ 10 μg As/L**	**< 10 μg As/L**	**OR (95% CI)**	**p-value**
High % Homeownership	28	233	.45 (.25, .72)	.002
Low % Homeownership	44	159		
High % People of Color	35	162	1.3 (.81, 2.2)	.3
Low % People of Color	37	230		

Fisher’s Exact test compares high and low categories of the variable of interest to CWSs whose average arsenic was above or below the revised MCL. The threshold for the variable of interest is determined by median value across all CWS, and includes related odds ratio. Test compares demographics for community water systems whose average arsenic was above or below the revised MCL.

#### Absolute measure of arsenic exposure

Results from the multivariate regression model examining the relationship between CWS demographics and absolute arsenic concentrations generally parallel descriptive findings. Unadjusted models had beta coefficients of −0.14 (95% Confidence Interval (CI), -0.34, 0.06) for homeownership, and −0.01 for percentage of people of color (95% CI, -.11, 0.08). Our adjusted model had a beta-coefficient of −0.27 (95% CI, -0.50, -0.05) for homeownership. This suggests that, on average, a 10% decrease in homeownership was associated with a 2.7 μg As/L increase, or roughly one third the mean arsenic concentration across all CWSs (6.0 μg As/L, see Table 1). The beta coefficient for percentage people of color was −0.02 (95% CI, -0.13, 0.09). This suggests that a 10% increase in the percentage of people of color served by a CWS was associated with an increase of .2 μg As/L, though this association was not statistically significant.

Results from our stratified model (Table 6) suggest similar, but stronger, trends among smaller systems. Among systems with less than 200 connections, the beta coefficient for homeownership was −0.43 (95% CI, -0.84, -0.03). This suggests that, on average, a 10% decrease in homeownership is associated with a 4.3 μg As/L increase, or nearly 70% of the mean arsenic concentration across all CWSs. The beta coefficient for percentage people of color was −0.17 μg As/L (95% CI, -0.36, 0.02), although this result was not statistically significant. In systems with at least 200 connections, the coefficients on percent homeownership and people of color were −0.19 (95% CI, -0.40, 0.02) and 0.03 (95% CI, -0.09, 0.15), respectively; neither of these results was statistically significant. Using this final stratified model to predict expected values, we estimated that arsenic levels in CWSs with 100% home ownership would be, on average, 3.1 μg As/L lower, compared to CWSs at the mean.

**Table 6:** **Regression**^**† **^**for factors associated with arsenic concentration (μg/L) in community water systems (CWS), 2005-2007, San Joaquin Valley, CA (n=464)**

**Variable**	**Model A**^**a**^	**Model B**^**a**^	**Model C**^**b**^	**Model D (< 200 Conections)**	**Model E (≥ 200 Connections)**
Constant	20.0 (6.7, 33.3)	11.2 (6.1, 16.4)	9.7 (−11.8, 31.3)	18.2 (−11.9, 49.1)	8.7 (−11.7, 49.1)
% People of Color		−0.01 (−0.11, 0.08)	−0.02 (−.13, 0.09)	−0.17* (−0.36, 0.02)	.03 (−0.09, 0.15)
% Home ownership	-.14 (−0.34, 0.05)		−0.27** (−0.50, -0.05)	−0.43** (−0.84, -0.03)	-.19* (−0.40, 0.02)
Groundwater or combined^c^			11.4*** (7.5, 15.2)	11.5*** (6.1, 16.9)	8.4*** (4.2, 12.6)
Private non-PUC regulated^d^			5.6* (−1.0, 12.2)	8.5** (0.73, 16.3)	1.2 (−5.4, 7.9)
Public^d^			6.9** (0.61, 13.11)	7.5* (−0.76, 15.8)	6.4* (−0.99, 13.8)
< 200 service connections			2.6 (−1.2, 6.5)	na	na
2006^e^			2.8** (0.52, 5.1)	4.4** (0.27, 8.4)	1.8 (−.76, 4.3)
2007^e^			1.2 (−0.51, 2.9)	2.4* (−0.11, 4.9)	.52 (−1.8, 2.9)
Summer/fall			-.27 (−1.9, 1.4)	.43 (−3.1, 4.0)	-.27 (−2.1, 1.5)
Valley ^f^			−1.4 (−6.5, 3.7)	6.4 (−2.3, 14.9)	−4.4 (−10.6, 1.8)
Foothills ^f^			6.9* (0.32, 13.5)	12.1*** (3.9, 20.4)	5.1 (−1.0, 11.3)

^†^Regression with robust standard errors, clustered by CWS. Coefficients represent the estimated difference in mean concentration at the system-level associated with a unit change in the covariate (95% CI); na=not applicable, as no CWSs in this model run contains this factor, or all CWSs have this factor.

^a^Unadjusted models, all CWSs included; ^b^Adjusted model, all CWSs included; ^c^ Surface water is referent category; combined refers to combination of groundwater and surface water sources; ^d^Privately owned PUC-regulated CWS as referent category; ^e^ 2005 is referent year; ^f^ Mountains is referent category.

* p < .10, ** p < .05, *** p < .01; R^2^ in Model B = .08; R^2^ in Model C = .09.

## Discussion

This study analyzed demographic differences in exposure and compliance burdens associated with the Revised Arsenic Rule in the San Joaquin Valley. We found that communities with lower rates of home ownership and greater proportions of people of color had higher odds of having an MCL violation. We also found a negative association between homeownership rates and arsenic concentrations in drinking water, with a stronger effect among smaller CWSs. These results indicate that communities with fewer economic resources faced a dual burden—they were not only exposed to higher arsenic levels, but were also served by systems more likely to receive an MCL violation.

Nearly 14% of the population in the study sample was potentially exposed to average arsenic levels above the revised standard, highlighting the health risks faced by Valley residents. At the revised level, cancer risks are estimated to be 12 in 10,000 and 23 in 10,000 for bladder cancer among women and men, respectively, and 18 in 10,000 and 14 in 10,000 for lung cancer, among women and men [12]. While we did not find a significant association between race/ethnicity and arsenic levels, a disproportionate number of the population potentially exposed to levels of 10 μg As/L or more were people of color. This indicates that as a whole, this group may still face disproportionate exposure.

Our results are consistent with previous findings that CWSs with higher arsenic levels serve customers with lower income levels [19]. Our results differ somewhat from a previous study [5] that found that while percent Latino was positively associated with the likelihood of exceeding the arsenic MCL, so was high SES. This difference could be due to differences in trends across states (i.e. Arizona vs. California), our additional measurements of exposure and compliance, or our focus on CWSs rather than all public water systems.

### Study limitations

Some limitations in our study are worth noting. As noted, the selection criteria we used (source location and arsenic samples) led to a slight under-representation of smaller systems in our final sample. Because the smallest systems had slightly higher arsenic levels and serve higher percentages of people of color and homeowners, this selection bias could also lead to an underestimate of our observed associations.

There are also several potential sources of measurement error in our dependent and independent variables. Under-reporting or under-issuing of violations could impact the count of MCL violations. However, sensitivity analyses comparing MCL violations in our final sample to results including all CWSs yielded consistent results. Similarly, sensitivity analyses comparing results using the binary MCL variable to binary measures that used average source-level concentrations were similar. Because of this consistent negative relationship between SES and each of these measures, we expect minimal impact on our results due to this potential under-reporting. This does not, however, explain why 41 CWSs (out of 72) had average system-level concentrations above the MCL but had no violation recorded; this may be related to selective enforcement and is worth further investigation.

There may also be some misclassification of points-of-entry into the distribution system. However, sensitivity analyses, including and excluding CWSs with treated and untreated point-of-entry sources yielded consistent regression coefficients for home ownership. While results for estimated exposure and compliance burdens are nearly five years old, we believe that, at a minimum, they capture current trends because unless CWSs have installed treatment plants or are using water from new wells (which is unlikely for small systems), temporal variability of arsenic levels is likely to be small [37]. Since our study focused only on CWSs, which excludes private well owners and communities with fewer than 15 service connections, the estimated number of potentially exposed people and impacted systems is likely to be an underestimate.

There may be errors in our demographic estimates, as we had to use data from the U.S. Census 2000 to approximate demographics between 2005 and 2007. There could also be error in our demographic estimates from: (1) surface intakes/well fields falling in Census block groups not served by the CWS, (2) not all Census block groups served by a CWS having an intake/field located within them, and (3) Latinos in Census data being undercounted due to legal status. For the majority of CWSs, sources fell within the same Census block groups that overlapped with the service area boundaries of CWSs [31]. But, because not all source/intake locations fell within block groups that intersect with service area boundaries of CWSs [31], this could lead to misclassification error of our demographic variables. This could result in a bias of the estimated association, but given the relatively small proportion of these systems, and the independence of SES status and inclusion in a linked census block group/water service boundary, this bias will be relatively trivial.

### Study implications

In California’s San Joaquin Valley, elevated arsenic levels are primarily derived from sedimentary deposits that can be mobilized by groundwater withdrawals and irrigation practices [10,11]. This means that our observed association could be partly explained by the location of low SES communities in relation to these agricultural activities. However, one would not necessarily expect a Valley-wide relationship between low SES and high arsenic levels, since arsenic is largely naturally occurring, and there are other areas in the Valley where low SES systems rely on shallower water.

Instead, our results can be understood as a reflection of the mediating role of system-level capacity. Smaller water systems often lack the economies of scale and resource-base to ensure the technical, managerial and financial (TMF) capacity to reduce contaminant levels [34,38]. They may be less able to install treatment, apply for funding, or drill new wells. The socioeconomic status of residents directly influences TMF capacity, because it affects the ability of a water system to leverage internal (e.g., rate increases) or external (e.g., loans) resources [34]. Thus, CWSs with lower SES customers may have been less able to support adequate TMF or to ensure compliance with the revised arsenic standard by 2007. That four of the six CWSs with treatment had more than 200 connections suggests that larger CWSs (with more resources and greater economies of scale) were able to comply more quickly with the revised standard, a result supported by previous research and acknowledged by the U.S. EPA [14,38]. Furthermore, that the majority of CWSs with average arsenic concentrations over the revised standard were small and had a high fraction of their wells with high arsenic levels indicates that these systems had few alternative sources of clean drinking water to begin with, making short-term solutions unattainable.

Our joint burden analysis highlights the need to consider not only exposure and current states of compliance, but also the *future* mitigation potential of impacted water systems and the households they serve. We have shown that CWSs with lower SES residents faced the greatest exposure and compliance burdens. Looking forward in time, these same systems may be the least equipped to comply with EPA drinking water standards for three reasons. First, these CWSs are often less able to develop long-term plans to reduce contamination. For example, some low SES communities in the Valley have secured funding to upgrade their infrastructure, but their plans failed to include steps to enter into compliance with the new arsenic standard [39]. Second, low-SES CWSs may be less able to apply for funding. By 2010, 13 of the 72 CWSs in our study with medium and high arsenic levels were not listed as having applied to the State Revolving Fund to help pay for mitigation options [40]. These CWSs were mainly small (< 200 connections) and had lower rates of home ownership (60% vs. 65%, p < .10) compared to CWSs that were listed. Funding sources, such as the State Revolving Fund, may further disadvantage small CWSs’ efforts to mitigate arsenic exposures and comply with the standard, because they require that systems have adequate TMF capacity to be eligible for funding. Finally, even with funding secured, low-SES water systems with low TMF capacity may be unable to maintain compliance. For example, some CWSs have installed arsenic treatment technologies, only to be forced to shut the plants down because they could not pay for ongoing treatment costs [41].

The combination of the low-SES of residents and low-TMF and compliance ability of CWSs not only impacts mitigation potential and exposure levels, it can also result in significant economic burdens for poorer households. In general, CWSs that are able to mitigate arsenic contamination will incur costs that are passed along to customers. Low-income residents find it hard to pay these higher rates, and may oppose mitigation efforts because of the impact on household budgets [42]. If a CWS cannot mitigate exposure, households may be forced to cope by buying bottled water, creating an additional economic burden. However, low-income residents may forgo such exposure-reduction measures, or only partially implement them [43]. In these cases, if a CWS remains in continuous non-compliance, chronic arsenic exposure risks will be prolonged.

Current debates regarding implementation of the Revised Arsenic Rule have discussed the option of using variances for small water systems, since the Safe Drinking Water Act allows for exemptions to meet compliance rules [23]. However, a short-term variance may only serve to create and perpetuate a two-tiered and inequitable system of regulation, in which low SES residents endure higher arsenic levels in their drinking water or are forced to rely on costly bottled water as an exposure reduction measure. Rather than variances, the regulatory system should provide targeted planning and technical support for small, disadvantaged communities to enter into compliance, so that provision of safe drinking water becomes logistically feasible. This support could include funding mechanisms to support regional system consolidation efforts that help small systems achieve economies of scale or draw on alternative water supplies.

## Conclusions

Using a “joint burden” approach, we examined the extent of exposure and compliance burdens in the San Joaquin Valley from 2005 to 2007. Our findings suggest that environmental justice concerns related to arsenic contamination in drinking water must consider both exposure and compliance burdens. Our work also highlights the need to better address how water systems serving low-SES residents can apply for and secure resources to enter into compliance, particularly if current funding criteria are tied to the technical, managerial and financial capacity of CWSs. That small, disadvantaged communities face greater compliance challenges highlights the need for appropriate regulatory measures and technical support. Ultimately, regional solutions that consolidate smaller CWSs serving economically disadvantaged communities within larger CWSs may be the best approach to addressing these disparities. In the interim, however, small water systems serving low SES residents will need enhanced funding and technical support to reduce community-level arsenic exposures.

## Abbreviations

As: Arsenic; CDPH: California Department of Public Health; CI: Confidence Interval; CWS: Community water system; GIS: Geographic Information System; IQR: Interquartile range; MCL: Maximum contaminant level; OR: Odds ratio; PEP: Population potentially exposed; PICME: Permits, Inspections, Compliance, Monitoring and Evaluation; PUC: Public utility commission; SDWA: Safe drinking water act; SES: Socioeconomic status; TMF: Technical, managerial and financial capacity; US EPA: United States environmental protection agency; μg As/L: Micrograms of arsenic per liter.

## Competing interests

The authors declare they have no competing interests.

## Authors’ contributions

CB conceived of the study and its design, acquired the data, performed statistical analyses and was the lead author of the article. RMF contributed to the design of the study, statistical analysis, and the writing of this article. AH contributed to the study design and statistical analysis. IR contributed to the design of the study and results analysis and played a significant role in the writing of the manuscript. All authors read, edited and approved the final manuscript.

## Supplementary Material

Additional file 1 **Table A1.** Shows a comparison of the initial population of active water systems, to the final sub-sample of systems. Click here for file

Additional file 2 **Figure A1.** Presents a schematic of a community water system that explains selection of point-of-entry sources. Click here for file

Additional file 3 **Table A2.** Presents results from two sets of sensitivity analyses using source-level average arsenic concentrations. Click here for file
