# Supplementary material for: Environmental justice implications of arsenic contamination in California’s San Joaquin Valley: a cross-sectional, cluster-design examining exposure and compliance in community drinking water systems
Source: Environ Health. 2012 Nov 14;11:84. doi: 10.1186/1476-069X-11-84 (PMC3533865; doi:10.1186/1476-069X-11-84)
Supplement: Additional file 1 — Table A1. Shows a comparison of the initial population of active water systems, to the final sub-sample of systems. [file 1476-069X-11-84-S1.doc]

Table A1. Characteristics of community water systems (CWSs) in study sample compared to all CWSs in study region with geographic coordinates. Study sample includes CWSs in San Joaquin Valley, CA (n=464), 2005-2007.

**Variable of Interest All active CWS in 2007 Active CWS CWS in study:**

**with and without with geographic active, with geographic geographic coordinates coordinates coordinates and samples**

**& with and without arsenic samples**

**n=671 n=644 n=464**

**Total population (count)** 3,064,936 3,037,785 1,134,017

**Population Characteristics (%)**

Latino population NA 43 46

Non-Latino People of Color NA 13 9

White population NA 47 45

Population above poverty^a^ NA 57 54

**Water System Characteristics (%)**

Mean People of Color NA 42 39

Mean Home Ownership NA 67 70

Population served (mean/median) 4,567/160 4,717 / 163 2,444 / 180

Incorporated ^b^  9 10 9

< 200 Connections 73 72 70

Groundwater Alone (GW)^c^ 86 88 92

GW and surface water^c^ 9 7 4

Publicly owned^d^ 32 32 32

Privately owned non-PUC reg.^d^ 60 60 61

**Water Quality Characteristics**

Min-Max; mean (µg As/L) NA NA 0-158; 6.0

IQR (µg As/L) NA NA 1.4, 6.3

CWS with ≥1 As MCL Viol NA 44 34

____________________________________________________________________________________________________________NA=not applicable because not all active sources had arsenic samples or geographic coordinates to estimate demographics; IQR= interquartile range.

^a^ Above 200% the poverty level; ^b^ A water system that serves a city that is a legally recognized municipal corporation with a charter from the state and governing officials that is incorporated, as opposed to a water system that serves an unincorporated area.^c^ Reference group=surface water only;15 unknowns; ^d^ Reference group=privately owned and Public Utility Commission (PUC) regulated or unknown.
