# Supplementary material for: Environmental justice implications of arsenic contamination in California’s San Joaquin Valley: a cross-sectional, cluster-design examining exposure and compliance in community drinking water systems
Source: Environ Health. 2012 Nov 14;11:84. doi: 10.1186/1476-069X-11-84 (PMC3533865; doi:10.1186/1476-069X-11-84)
Supplement: Additional file 2 — Figure A1. Presents a schematic of a community water system that explains selection of point-of-entry sources. [file 1476-069X-11-84-S2.docx]

Additional File 1, Figure A1. Schematic of a community water system (CWS). Schematic indicates: a) that water from a groundwater well or stream may be treated or untreated before entering into the distribution system, b) location of point-of-entry sources and c) use of proxy for tap water quality.

In a system with untreated sources, water entering the distribution may flow from a groundwater well (point a), or from a surface water source (point b). In a system with no treatment, if points a and b flow into the same point-of-entry (point f), the average arsenic levels of each source is averaged at point f. If points a and b do not flow into a common point-of-entry, each is, in essence, a point-of-entry. In a system *with* treatment, water from surface water (point c) or groundwater sources (point d) is treated for arsenic at a treatment facility, and point e is the common point of entry. The average concentration at all point-of-entry sources is used to represent average water quality in the distribution system (point g), which is a proxy for tap water quality (point h).
