# Supplementary material for: Environmental justice implications of arsenic contamination in California’s San Joaquin Valley: a cross-sectional, cluster-design examining exposure and compliance in community drinking water systems
Source: Environ Health. 2012 Nov 14;11:84. doi: 10.1186/1476-069X-11-84 (PMC3533865; doi:10.1186/1476-069X-11-84)
Supplement: Additional file 3 — Table A2. Presents results from two sets of sensitivity analyses using source-level average arsenic concentrations. [file 1476-069X-11-84-S3.doc]

Table A2. Fisher’s Exact Tests used in sensitivity analysis to compare source-level averages instead of MCL violations. Two sensitivity analyses were run. The first was based on whether at a system-level any source’s yearly average was greater than the maximum contaminant level (MCL). The second was based on whether at a system-level any source’s average arsenic concentration during the entire study period (i.e. compliance period 2005-2007) exceeded the MCL. Results from both tests are consistent with each other and with results shown in Table 4 of main document.

| **Sensitivity Analysis Variable of Interest** ≥ **10 µg As/L <10 µg As/L** **OR (95% CI) p-value** |
| --- |
| *Source yearly*  *average >MCL* |
| High % Homeownership 29 232 .40 (.24, .66) .0004 |
| Low % Homeownership 48 155 |
|  |
| High % People of Color 41 156 1.7 (1.03, 2.7) .04 |
| Low % People of Color 36 231 |
| *Source study period*  *average >MCL* |
| High % Homeownership 27 234 .38 (.23, .64) .0003 |
| Low % Homeownership 47 156 |
|  |
| High % People of Color 40 157 1.7 (1.1, 2.8) .03 |
| Low % People of Color 34 233 |
